# Buckwheat Hull-Enriched Pasta: Physicochemical and Sensory Properties

**DOI:** 10.3390/molecules27134065

**Published:** 2022-06-24

**Authors:** Katarzyna Sujka, Grażyna Cacak-Pietrzak, Alicja Sułek, Karolina Murgrabia, Dariusz Dziki

**Affiliations:** 1 Department of Food Technology and Assessment, Institute of Food Sciences, Warsaw University of Life Sciences, 159C Nowoursynowska Street, 02-776 Warsaw, Poland; katarzyna_sujka@sggw.edu.pl (K.S.); grazyna_cacak_pietrzak@sggw.edu.pl (G.C.-P.); 2 Department of Cereal Crop Production, Institute of Soil Science and Plant Cultivation—State Research Institute, 8 Czartoryskich Street, 24-100 Puławy, Poland; sulek@iung.pulawy.pl; 3 Faculty of Food Technology, Warsaw University of Life Sciences, 159C Nowoursynowska Street, 02-776 Warsaw, Poland; k.murgrabia@gmail.com; 4 Department of Thermal Technology and Food Process Engineering, Lublin University of Life Sciences, Głęboka 31 Street, 20-612 Lublin, Poland

**Keywords:** wheat, buckwheat hull, chemical composition, cooking, antioxidant activity, texture

## Abstract

This work aimed to evaluate the effect of partial replacement of semolina with 0, 1, 5, 10, 15, and 20% of ground buckwheat hull (BH) on the chemical composition, antioxidant properties, color, cooking characteristics, and sensory properties of wheat pasta. Pasta samples were prepared by dough lamination (tagliatelle shape) and dried at 55 °C until the moisture content was 11–12% (wet basis). Analyses of samples showed that the addition of BH caused an increase in fiber content in pasta from 4.31% (control pasta) to 14.15% (pasta with 20% of BH). Moreover, the brightness and yellowness of BH-enriched products were significantly decreased compared to the control sample, and the total color difference ranged from 23.84 (pasta with 1% of BH) to 32.56 (pasta with 15% BH). In addition, a decrease in optimal cooking time, as well as an increased weight index and cooking loss, was observed in BH-enriched pasta samples. Furthermore, BH-enriched cooked pasta had significantly higher total phenolic content and antioxidant activity but an unpleasant smell and taste, especially if the level of BH was higher than 10%.

## 1. Introduction

Wheat pasta (WP) is usually prepared using semolina and water and preserved by drying. This product is popular among consumers mainly due to its short time and ease of preparation. WP is also energy-dense, low in sodium and fat with no cholesterol, and a common ingredient in many dishes. Classic pasta is characterized by a high content of starch, with minor amounts of dietary fiber, vitamins, and minerals; however, its nutritional and health value can be improved by enrichment with different raw materials [1]. In particular, over the past few years, various plant by-products, which are a cheap source of fiber and biologically active compounds, have been added to WP [2]. Schettino et al. [3] attempted partial replacement of semolina with brewers’ spent grain in order to improve the chemical composition and antioxidant properties of pasta. Other authors aimed to increase the antioxidant potential and nutritional value of WP by adding by-products from the oil industry [4], fruit and vegetable industry [5,6,7], and even from the animal industry [8,9,10]. These additives contributed to increasing the nutritive value and health properties of WP but often had a negative effect on the cooking and sensory characteristics of the product [11]. Thus, the production of high-quality pasta from nonconventional raw materials is a challenge and the level of addition of each ingredient should be optimized for better results.

A valuable ingredient for pasta fortification can be buckwheat and especially buckwheat hull (BH). Buckwheat is a pseudocereal belonging to the genus *Fagopyrum*. The most predominant buckwheat species is the common buckwheat (*Fagopyrum esculentum Moench*) [12]. Buckwheat is characterized by good adaptability and hence can grow in different environments [13]. Its seeds contain various nutrients such as carbohydrates, proteins, phenolics, vitamins, carotenoids, minerals, and phytosterols [14]. Buckwheat flour is widely used in the production of noodles, pancakes, and pasta [15,16] or for husking [17]. BH accounts for about 22% of the total mass of processed grain [18]. It is rich in fiber, bioactive compounds, and flavonoids especially rutin (0.5–0.7%) [16] and exhibits high antioxidant activity (AA). Rutin (3,3′,4′,5,7-pentahydroxyflavone-3-rhamnoglucoside) is a flavonol commonly found in plants, which has strong antioxidant, neuroprotective, anticarcinogenic, vasoprotective, cytoprotective, and cardioprotective properties [19]. Other flavonoids such as quercetin, isoquercetin, isovitexin, vitexin, orientin and iso-orientin, and hyperoside are also found in common BH [20,21]. Thus, BH can be used as a food ingredient [22,23,24] and also many potential applications in medicine [25], biotechnology [26], and textile industry [27]. It is also used as a filler for pillows and blankets, as a packaging material for fruits, and as fuel and feed [18].

Buckwheat flour is used as the main ingredient of buckwheat pasta. A recent study showed that good-quality noodles can be produced by mixing 75% of buckwheat flour and 25% of common wheat flour [28]. Another study [15] investigated the possibility of partial replacement of wheat flour with BH and common buckwheat bran (up to 5%) for the production of wheat noodles. However, the effect of the addition of BH to durum WP has not been analyzed so far. The present work aimed to study the effect of partial replacement of semolina by BH on the quality, chemical composition, antioxidant activity, and sensory properties of WP.

## 2. Results and Discussion

### 2.1. Basic Chemical Composition of Raw Materials and Pasta

The basic composition of semolina, BH, and pasta is shown in Table 1. The analysis of chemical composition showed that semolina had about twofold higher content of protein compared to BH but similar content of fat. On the other hand, BH had higher amount of ash and about 19-fold higher content of fiber than semolina. A similar composition of BH has been reported by other authors [29]. Enriched pasta prepared by partial replacement of semolina with BH had slightly decreased protein content, ranging from 11.69% (B1) to 10.50% (B20). Furthermore, addition of buckwheat caused a significant increase in fiber content in pasta. Control pasta (CP) was characterized by similar fiber content as semolina (about 4.31%), while B20 had 14.15% of fiber. Moreover, as the mass fraction of BH in the recipe increased, the ash content in pasta also increased. The fat content in both raw materials and enriched pasta was found to be at a similar level (0.51% in average).

### 2.2. Color of Pasta Samples

Color is one of the most important quality determinants of food. The color of pasta depends on the raw materials used [30] and technological process [31]. For durum WP, brightness and yellowness are considered important parameters by both consumers and manufacturers. A bright yellow color indicates that the pasta is of high quality [32]. However, enrichment of pasta often leads to color changes [33,34]. In this study, the addition of BH to semolina caused a significant decrease in the lightness of pasta from 65.27 (CP1) to 39.14 (B20). Importantly, replacement of semolina with 1% of BH caused the highest decrease of L* (from 65.27 to 45.72) (Table 2). A similar tendency was found for the yellowness parameter (b*), with the highest values estimated for CP (18.97). Enrichment with BH also caused a significant decrease in yellowness from 5.34 (B1) to 0.48 (B20). On the other hand, addition of BH had a relatively slight influence on the redness (a*) of the product. The highest a* value was found for B5 (2.37) and the lowest for B15 (0.85). Interestingly, the redness of the control sample (1.38) did not significantly differ from that of the B20 (1.16) sample (*p* > 0.05). Changes in the color of pasta are caused by changes in pigment content in BH. The most predominant pigment in BH is melanin which is localized in the outer layers of BH cell walls [18]. Melanin is a high-molecular-weight pigment formed by the oxidation and polymerization of phenolic compounds. It has been shown to protect cells against carcinogenic and mutagenic factors [35]. In this study, the total color difference (ΔE) in enriched samples ranged from 23.84 (B1) to 32.56 (B15). Interestingly, the addition of BH at a level of 10% and higher had only a slight influence on ΔE. This suggests that BH pigments have a strong influence on the color of the pasta, and its addition at even 10% can cause saturation of the product’s color. The highest ΔE value was observed for the B1 sample. The color changes between the samples were clearly visible if ΔE was >3.5 [36].

### 2.3. Cooking Quality of Pasta 

The optimal cooking time (OCT) of pasta was dependent on the level of BH in the recipe (Table 3). Addition of BH reduced OCT from 4.8 min (CP) to 3.7 min (B20). OCT is influenced by various factors such as the raw materials used for pasta preparation [37], the parameters of the technological process [37], and the type of pasta [38]. Some authors found a positive correlation between the total dietary fiber content in pasta and OCT [39,40]. By contrast, a negative relationship was found in the present study and in others [33,41]. This suggests that different sources of fiber and ratios of soluble to insoluble fiber can have varied effects on the cooking properties of pasta. At a high amount, fiber often negatively influences the cooking properties of pasta due to the disruption of the starch–protein matrix dough during dough mixing and can swell rapidly during pasta cooking [40]. On the other hand, some nontraditional ingredients with high fiber content can have no negative effect on the quality of pasta or even increase its cooking quality [2,42].

An important cooking property that determines the texture of pasta is its ability to increase weight. Thus, the weight increase index (WII) parameter is determined by authors. In the present study, significantly (*p* < 0.05) higher WII was found for B15 and B20 samples compared to CP, while the values determined for CP, B1, B5, and B10 samples did not statistically differ (Table 3). It must be mentioned that WII depends on the ingredients used for pasta preparation [2], pasta cooking time [43], and the method used for pasta drying [44].

Cooking loss (CL) determined for control and enriched pasta is presented in Table 3. In general, CL should not be higher than 8% for pasta made from semolina [45]. In this study, enrichment with BH resulted in increased CL in pasta and the values ranged from 7.0% dry matter (DM) (CP) to 10.7% DM (B20). However, for the other BH-enriched pasta samples, CL did not exceed 8.1% DM. Recently, Liu et al. [15] found similar CL in noodles made from common wheat flour with up to 5% BH. CL of pasta depends mainly on cooking time [39], cooking method [46], and raw materials used for production. Addition of different plant additives to WP has been shown to result in higher CL [42,43]. Lower CL indicates lower loss of nutrients [45]. Higher CL observed for enriched pasta in this study may be attributed to weakening of gluten resulting from the addition of BH to semolina. Partial replacement of semolina by fiber-rich raw materials often results in an increase in CL. Sobota et al. found that the addition of wheat bran (from 20% to 40%) to semolina caused a linear increase in CL [47].

### 2.4. Total Phenolic Content and Antioxidant Activity

The total phenolic content (TPC) of BH was estimated at 21.55 mg gallic acid equivalent (GAE)/g DM which was about 20-fold higher compared to semolina (Table 4). Consequently, the AA of BH was significantly higher (*p* < 0.05) compared to semolina. AA was expressed as the value of EC_50_ (half-maximal inhibitory concentration that causes a 50% decrease in activity). The EC_50_ of BH extracts for ABTS and DPPH was respectively 2.96 and 8.13 mg DM/mL, whereas the value determined for semolina was significantly (*p* < 0.05) and relevantly lower (93.36 mg and 60.31 mg DM/mL, respectively). Li et al. [48] estimated the TPC of different parts of buckwheat seeds and found that BH was characterized by the highest content of both free and bound phenolics compared to buckwheat bran and flour. Importantly, free phenolics were predominant in BH, bran, and flour. Other authors have also confirmed that BH is an excellent source of phenolic and antioxidant compounds, mainly anthocyanins, flavanones, flavanol, flavones, flavonols, and phenolic acids [49]. Recently, Noore et al. [50] identified 41 phenolic compounds in BH. BH is especially rich in phenolics such as protocatechuic acid, hyperoside, vitexin, isovitexin, and rutin (in increasing order) [51]. Thus, in the present study, BH-enriched pasta had a significantly (*p* < 0.05) higher level of phenolics than CP. The TPC of cooked pasta ranged from 1.09 mg GAE/g DM (CP) to 2.54 mg GAE/g DM (B20). After the addition of BH, the AA of pasta against both DPPH and ABTS increased. The lowest values of EC_50_ or the highest AA were found for B20 (34.22 and 37.40 mg GAE/g DM for DPPH and ABTS, respectively), whereas the lowest AA for CP (112.62 and 64.29 for DPPH and ABTS, respectively) (Table 4). Addition of raw materials that are rich in fiber to semolina or wheat flour leads to an increase in the AA of pasta [2]. Sinkovič et al. [52] found that BH obtained from common buckwheat has higher AA expressed as DPPH compared to whole buckwheat grain, whereas lower AA was observed for Tartary buckwheat.

### 2.5. Results of Pasta Sensory Evaluation

**Figure 1:**
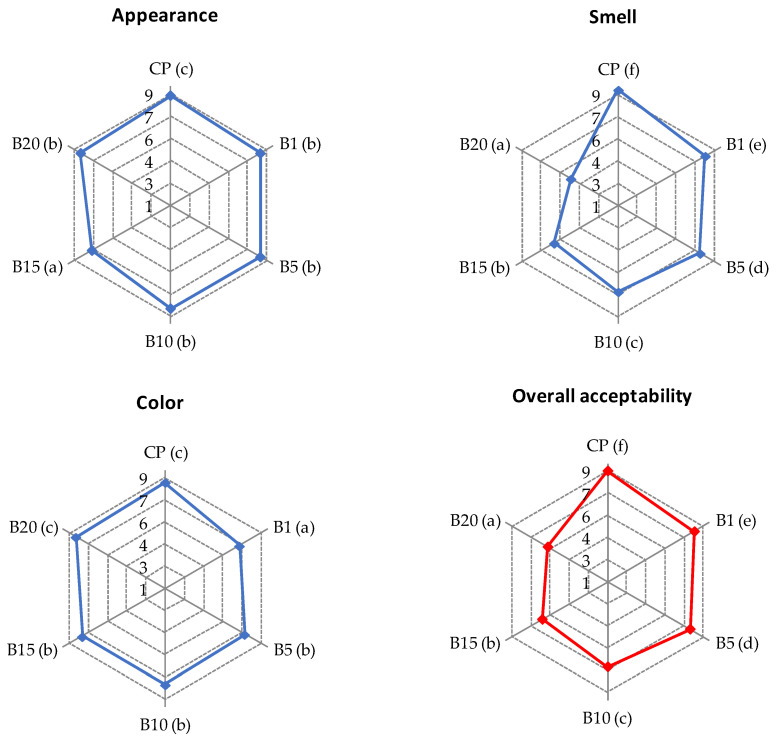
Results of sensory evaluation of raw pasta: CP—control pasta; B1, B5, B10, B15, and B20—pasta with 1, 5, 10, 15, and 20% of BH, respectively. Values of each parameter with different superscript letters in the brackets are significantly different at *p* < 0.05.

**Figure 2:**
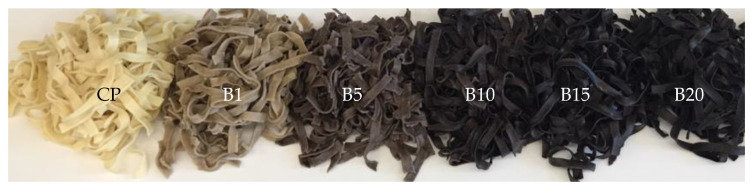
Cooked pasta samples: CP—control pasta; B1, B5, B10, B15, and B20—pasta with 1, 5, 10, 15, and 20% of BH, respectively.

**Figure 3:**
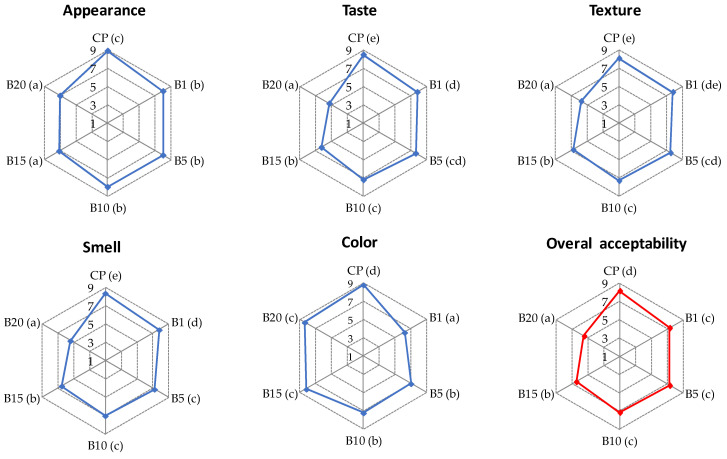
Results of sensory evaluation of cooked pasta: CP—control pasta; B1, B5, B10, B15, and B20—pasta with 1, 5, 10, 15, and 20% of BH, respectively. Values of each parameter with different superscript letters in the brackets are significantly different at *p* < 0.05.

Sensory evaluation showed that the addition of BH to semolina caused significant (*p* < 0.05) changes in the appearance, smell, color, and consequently overall acceptability of raw pasta (Figure 1). The highest scores for these attributes were achieved by CP, while BH enrichment resulted in decreased scores for all quality properties of pasta. In particular, enriched pasta samples obtained lower scores for smell. The smell of samples with 15% and 20% of BH (B15 and B20, respectively) was assessed as liked slightly and neither liked nor disliked, respectively, as a result of which these samples received the lowest scores for overall acceptability. CP was assessed as liked very much, whereas B20 was rated as neither liked nor disliked. Interestingly, CP and B20 received the highest scores for color, whereas the lower scores were obtained by pasta with 1% of BH (B1). This suggests that the panelists preferred pasta with both light and dark colors. A similar tendency was found for cooked pasta (Figure 2 and Figure 3). The appearance of all pasta samples was highly acceptable, whereas the lowest scores for smell and taste were found for B15 and B20 samples. Replacement of semolina with BH at a level higher than 10% resulted in an unpleasant smell and slightly spicy taste. The unpleasant flavor can be attributed to the high tannin content of BH [18]. Except for B1, all pasta samples received high scores for color. Interestingly, the brightest (CP) and darker pasta (B15 and B20) samples received the highest scores for the color parameter. Similar results were found in a study in which carob flour was added to WP [33]. On the other hand, the B1 sample was assessed as neither liked nor disliked as a result of its brown color. CP was assessed as highly acceptable based on its texture, whereas B20 was assessed by most of the panelists as slightly liked. With the increase in the percentage of BH in the pasta recipe, cooked samples were less firm and more sticky. However, B1, B5, and B10 were assessed as highly acceptable by the panelists. Taking into account the overall acceptability of enriched pasta, the level of BH added to semolina should not exceed 10%. A similar result was reported by other authors for the addition of wholegrain native buckwheat flour to WP [53]. 

## 3. Materials and Methods

### 3.1. Materials

Semolina (*Triticum durum* L.) was purchased from Polskie Młyny (Warsaw, Poland), and BH from BioPlanet (Leszno, Poland).

The basic chemicals used in the study as well as Folin–Ciocalteu reagent, ABTS (2,2′-azino-bis-(3-ethylbenzothiazoline-6-sulphonic acid), DPPH (2,2-diphenyl-1-picrylhydrazyl), and methanol and sodium carbonate were purchased from Sigma-Aldrich Company (Poznan, Poland).

### 3.2. Pasta Production

Pasta samples were prepared from semolina (CP) or from the mixtures of semolina and ground BH (particle size < 250 µm). Semolina was replaced with 1, 5, 10, 15, or 20% of BH, and 35% of water was added to semolina and semolina–BH mixtures. Dough was mixed for 5 min using a mixer (model T-5KPM5EER, KitchenAid, Greenville, SC, USA). Then, the dough was rolled into 2-mm-thick sheets and laminated (tagliatelle shape) using a pasta rolling device (5KSMPSA, KitchenAid, Greenville, SC, USA). All samples were dried at 55 °C in a compartment dryer for 6 h. The moisture content of pasta ranged between 11 and 12% (wet basis) after drying. Each sample was prepared in triplicate.

### 3.3. Chemical Composition of Raw Materials and Pasta

The basic chemical composition of raw materials (semolina and BH) and pasta samples were characterized according to the AACC methods [54]. The components were analyzed: moisture content (AACC, Method 44-15.02), ash content (AACC, Method 08-01.01), protein content (AACC, Method 46-10.01), fat content (AACC, Method 30-10.01), and total dietary fiber content (AACC, Method 32-05.01). The content of carbohydrates in both raw materials and pasta samples was determined based on the difference from these values [39]. 

### 3.4. Cooking Quality of Pasta

The OCT of pasta samples was assessed as described by Zarzycki et al. [55]. Briefly, 100 g of pasta sample was added to 1 L of boiling distilled water. The sample was gently boiled at a power of 600 W using an induction cooker (TCL 34P2, Huizhou, China). Then, the pasta was squeezed between two plates of glass every 30 s. OCT was calculated as the time after which no white central core of the sample can be observed.

WII was calculated by dividing the weight of the pasta sample after cooking by the weight of an uncooked pasta sample (100 g) [40].

CL or loss of pasta dry mass during cooking was determined on the basis of the liquid remaining after pasta boiling. Briefly, l00 g of pasta sample was added to 1 L of boiling water and cooked on a low heat in accordance with the predetermined OCT. After boiling, the liquid was cooled to room temperature and its volume was measured in a graduated cylinder. Then, the liquid was mixed and 25 mL of it was transferred to a weighing vessel. The vessel was dried in an oven to constant weight at 105 °C. After cooling, the vessel was weighed with an accuracy of 0.01 g. CL was calculated as a percentage using the following formula:

(1) $$\mathrm{CL}=\left[\frac{\mathrm{Vc}}{\mathrm{Vp}}\times \frac{\left(m_{p}-m_{a}\right)}{m_{d}}\right]\times 100\%\;$$

where v_c_ is the total volume of liquid after pasta cooking, v_p_ is the volume of liquid taken for drying, m_p_ and m_a_ are respectively the mass of the vessel with the sample before and after drying, and m_d_ is the content of dry mass in the pasta before drying.

### 3.5. Pasta Color

The color of pasta samples was determined using a Konica Minolta spectrophotometer (CM-3600d, Tokyo, Japan) in the CIE L*a*b* (CIELAB) color space with a D65 illuminant and 10° observer as a reference system. The following color parameters were determined: lightness (L*), a (positive value indicates redness and negative indicates greenness), and b* (positive value indicates yellowness and negative indicates blueness). The total color difference (ΔE) between the color of control and enriched samples was calculated from the following formula [36]:

(2) $$\Delta E=\sqrt{{\left(\Delta L^{*}\right)}^{2}-{\left(\Delta a^{*}\right)}^{2}-{\left(\Delta b^{*}\right)}^{2}}$$

The measurements were performed five times for each pasta sample.

### 3.6. Total Phenolic Content and Antioxidant Activity

#### Extract Preparation

Methanolic extracts (methanol:water, 1:1, *v*/*v*) of pasta samples were prepared for determining their TPC and AA [56]. TPC was determined using the Folin–Ciocalteu method [36]. Briefly, 0.5 mL H_2_O, 0.5 mL of the sample, and 2 mL of Folin–Ciocalteu reagent (1:5 H_2_O) were mixed. After 3 min, 10 mL of 10% Na_2_CO_3_ was added. Absorbance of samples was read after 30 min at 725 nm using a UV–Vis spectrophotometer (UV-1900 UV-VIS, Shimadzu, Osaka, Japan). The amount of TPC was calculated and expressed in milligrams GAE per gram DM.

Antioxidant activity against DPPH and ABTS radicals was determined using a plate spectrophotometer (Model Epoch2TC, S/N 15120115, Aligent, BioTek, Santa Clara, CA, USA). The ABTS radical scavenging activity was determined as described by Re et al. [57], with slight modifications [58]. Briefly, ABTS+^•^ radicals were generated by the oxidation of ABTS using potassium persulfate (PP) as an oxidizing agent. The ABTS radical cation was obtained by mixing 7 mmol/L stock solution of ABTS with 2.45 mmol/L PP. The resulting solution was diluted with distilled water until an absorbance of 0.7 ± 0.05 at 734 nm was reached. Then, 40 μL of the extract was added to 1.8 mL of ABTS+^•^ solution, and the absorbance was measured at intervals of 5 min. The ability of the extracts to quench the ABTS free radical was determined using the following formula:

(3) $$\mathrm{Scavenning}\;\mathrm{activity}\;=\frac{A_{c}-A_{s}}{A_{c}}\times 100\;\%,$$

where A_s_ is the absorbance of the sample and A_c_ is the absorbance of the control.

The DPPH radical scavenging activity was determined as described by Brand-Williams et al. [59]. Briefly, 40 μL of the extract was mixed with 3.92 mL of 6 × 10^–5^ mol/L solution of DPPH• in methanol. Absorbance was measured after 10 min of incubation. The ability of the extract to quench DPPH free radical was determined according to Equation (1). The antiradical activity of samples against ABTS and DPPH was expressed as half-maximal inhibitory concentration (EC_50_), which refers to the concentration that causes a 50% decrease in activity.

### 3.7. Sensory Evaluation of Pasta

Sensory evaluation was performed on both raw and cooked pasta samples. The samples were rated on a 9-point hedonic scale, with scores ranging from 1 (dislike extremely) to 9 (like extremely), as per a previously described method [60]. All pasta samples were evaluated for appearance, smell, color, taste, texture, and overall acceptability. Taste and texture were assessed only for cooked samples. The evaluation was carried out by an untrained team of 52 panelists who commonly consume WP. Analysis was performed under white lighting and at a constant temperature of 21 °C. All panelists were asked to drink water before testing each sample.

### 3.8. Statistical Analysis

All analyses were performed in a minimum of three replicates. The results were statistically evaluated in Statistica 13.3 software (TIBCO Software, Palo Alto, CA, USA). Comparisons among groups were carried out using a one-way analysis of variance, while homogeneous groups were compared using Tukey’s test. A significance level of α = 0.05 was fixed for the analysis.

## 4. Conclusions

Enrichment of WP with additional fiber is a current trend that contributes to improving both the nutritional and health attributes of the product. In particular, by-products from the food industry which are a cheap and valuable source of fiber can be used for this purpose [2]. This study demonstrated that the addition of BH to food matrices such as pasta causes significant changes in the physicochemical properties of products. The results of the analyses of samples indicated that there was an increase in dietary fiber and ash content whereas the content of protein was decreased due to the addition of BH to semolina. BH enrichment also caused significant color changes in pasta, especially a decrease in lightness and yellowness parameters. Moreover, the cooking properties of pasta were significantly influenced by BH. A decrease in OCT was observed, but WII and CL increased with increasing content of BH in the pasta recipe. Sensory evaluation of both raw and cooked pasta samples showed that replacement of semolina with BH had a negative effect on pasta smell and taste. On the other hand, BH-enriched pasta had higher phenolic content and enhanced AA. The results of the study suggest that BH-enriched pasta can be a good alternative for people seeking healthy food with increased fiber content and higher antioxidant potential. However, the level of BH added to semolina should not exceed 10%. Pasta containing BH has acceptable cooking and sensory properties with about twofold higher TPC compared to BH-free pasta.

## Figures and Tables

**Table 1:** Basic composition [%] of raw materials and pasta.

Sample	Moisture	Protein	Ash	Fat	Fibre	Carbohydrates
SE	10.29 ± 0.07 ^A^	11.70 ± 0.21 ^B^	0.88 ± 0.01 ^A^	0.51 ± 0.01 ^A^	4.31 ± 0.04 ^A^	74.62
BH	8.22 ± 0.06 ^B^	5.40 ± 0.02 ^A^	1.93 ± 0.02 ^B^	0.50 ± 0.02 ^A^	78.87 ± 0.70 ^B^	83.95
CP	10.33 ± 0.05 ^a^	11.73 ± 0.15 ^d^	0.87 ± 0.01 ^a^	0.52 ± 0.02 ^a^	4.27 ± 0.08 ^a^	76.55
B1	10.49 ± 0.04 ^ab^	11.69 ± 0.03 ^d^	0.90 ± 0.02 ^ab^	0.52 ± 0.01 ^a^	4.62 ± 0.03 ^a^	76.41
B5	10.34 ± 0.05 ^a^	11.30 ± 0.02 ^c^	0.93 ± 0.02 ^b^	0.52 ± 0.01 ^a^	7.51 ± 0.05 ^b^	76.91
B10	10.40 ± 0.12 ^ab^	11.04 ± 0.04 ^b^	1.00 ± 0.01 ^c^	0.50 ± 0.02 ^a^	9.86 ± 0.06 ^c^	77.07
B15	10.44 ± 0.05 ^ab^	10.87 ± 0.03 ^b^	1.05 ± 0.02 ^d^	0.51 ± 0.01 ^a^	11.89 ± 0.04 ^d^	77.13
B20	10.58 ± 0.15 ^b^	10.50 ± 0.02 ^a^	1.12 ± 0.02 ^e^	0.49 ± 0.01 ^a^	14.15 ± 0.08 ^e^	77.31

SE—semolina; BH—buckwheat hull; CP—control pasta; B1, B5, B10, B15, and B20—pasta with 1, 5, 10, 15, and 20% of BH, respectively. Data are presented as mean (*n* = 3) with standard deviation. Values of each parameter with different superscript letters are significantly different at *p* < 0.05.

**Table 2:** Color coordinates of control and enriched pasta.

Sample	L*	a*	b*	ΔE
CP	65.27 ± 0.07 ^e^	1.38 ± 0.09 ^c^	18.97 ± 0.24 ^d^	-
B1	45.72 ± 0.06 ^d^	2.09 ± 0.04 ^d^	5.34 ± 0.14 ^c^	23.84
B5	43.27 ± 0.23 ^c^	2.37 ± 0.15 ^d^	3.40 ± 0.40 ^b^	26.97
B10	38.72 ± 0.38 ^b^	1.09 ± 0.01 ^ab^	0.91 ± 0.28 ^a^	32.11
B15	36.68 ± 0.52 ^a^	0.85 ± 0.18 ^a^	0.74 ± 0.01 ^a^	32.56
B20	39.14 ± 0.03 ^b^	1.16 ± 0.03 ^bc^	0.48 ± 0.02 ^a^	31.99

L*—lightness; a*—redness; b*—yellowness; ΔE—total color difference; CP—control pasta; B1, B5, B10, B15, and B20—pasta with 1, 5, 10, 15, and 20% of BH, respectively. Data are presented as mean (*n* = 5) with standard deviation. Values of each parameter with different superscript letters are significantly different at *p* < 0.05.

**Table 3:** Cooking properties of pasta.

Sample	OCT [min]	WII [–]	CL [% DM]
CP	4.8 ± 0.1 ^e^	2.7 ± 0.1 ^bc^	7.0 ± 0.1 ^a^
B1	4.5 ± 0.1 ^d^	2.5 ± 0.1 ^ab^	7.6 ± 0.1 ^b^
B5	4.4 ± 0.1 ^cd^	2.6 ± 0.1 ^ab^	7.9 ± 0.0 ^c^
B10	4.2 ± 0.0 ^bc^	2.7 ± 0.1 ^bc^	8.1 ± 0.1 ^c^
B15	4.1 ± 0.0 ^b^	2.8 ± 0.1 ^c^	8.1 ± 0.1 ^c^
B20	3.7 ± 0.0 ^a^	3.0 ± 0.2 ^d^	10.7 ± 0.2 ^d^

OCT—optimal cooking time; WII—weight increase index; CL—cooking loss; DM—dry mass, CP—control pasta; B1, B5, B10, B15, and B20—pasta with 1, 5, 10, 15, and 20% of BH, respectively. Data are presented as mean (*n* = 3) with standard deviation. Values of each parameter with different superscript letters are significantly different at *p* < 0.05.

**Table 4:** Total phenolic content and antioxidant activity of raw materials and pasta.

Sample	TPC [mg GAE/g DM]	EC_50__DPPH_ [mg DM/mL]	EC_50__ABTS_ [mg DM/mL]
SE	1.06 ± 0.03 ^A^	93.36 ± 1.07 ^B^	60.31 ± 0.82 ^B^
BH	21.55 ± 0.23 ^B^	2.96 ± 0.09 ^A^	8.13 ± 0.31 ^A^
CP	1.09 ± 0.04 ^a^	112.62 ± 1.94 ^f^	64.29 ± 1.03 ^e^
B1	1.20 ± 0.03 ^b^	102.29 ± 0.67 ^e^	59.37 ± 1.56 ^d^
B5	1.48 ± 0.05 ^c^	70.37 ± 1.16 ^d^	52.45 ± 1.05 ^c^
B10	1.98 ± 0.03 ^d^	44.52 ± 0.57 ^c^	47.12 ± 1.23 ^b^
B15	2.26 ± 0.06 ^e^	39.67 ± 0.81 ^b^	42.36 ± 3.80 ^a^
B20	2.54 ± 0.11 ^f^	34.22 ± 0.96 ^a^	37.40 ± 2.31 ^a^

TPC—total phenolics content, EC_50DPPH_—antiradical activity against DPPH, EC50_ABTS_ —antiradical activity against ABTS, DM—dry mass, SE—semolina; BH—buckwheat hull; CP—control pasta; B1, B5, B10, B15, and B20—pasta with 1, 5, 10, 15, and 20% of BH, respectively. Data are presented as mean **(***n* = 3) with standard deviation. Values of each parameter with different superscript letters are significantly different at *p* < 0.05.

## Data Availability

The data presented in this study are available on request from the corresponding author.

## Sample Availability

Not available.
